# Recurrent thoracic duct cyst of the left supraclavicular fossa: A retrospective study of 6 observational case series and literature review

**DOI:** 10.1097/MD.0000000000028213

**Published:** 2021-12-17

**Authors:** Julie Planchette, Clara Jaccard, Audrey Nigron, Jean-Baptiste Chadeyras, Guillaume Le Guenno, Benjamin Castagne, Yvan Jamilloux, Anne-Sophie Resseguier, Pascal Sève

**Affiliations:** a Department of Internal Medicine and Rheumatology, Hospital Emile Roux, Le Puy en Velay, France; b Department of Radiology, Hopital Emile Roux, Le Puy en Velay, France; c Department of Thoracic Surgery, Hospital Jean Perrin, Clermont-Ferrand, France; d Department of Internal Medicine, CHU Estaing, Clermont-Ferrand, France; e Department of Internal Medicine, Lyon University Hospital, Lyon, France; f Université Claude Bernard Lyon 1, Research on Healthcare Performance (RESHAPE), INSERM Lyon, France.

**Keywords:** high-fat diet, lymphocele, supraclavicular fossa, thoracic duct cyst, thoracic duct lymphangiectasia

## Abstract

The transient occlusion of the terminal thoracic duct is a rare disease responsible for renitent supraclavicular cysts. The aim of this study was to describe the clinical characteristics, evolution, and treatment.

A retrospective multicenter study and literature review was carried out. The literature search (PubMed) was conducted including data up to 31 December 2020 and PRISMA guidelines were respected.

This study identified 6 observational cases between September 2010 and December 2020. The search results indicated a total of 24 articles of which 19 were excluded due to the lack of recurrent swelling or the unavailability of full texts (n = 5). Fourteen patients (8 from literature) mostly reported a noninflammatory, painless renitent mass in the supraclavicular fossa which appeared rapidly over a few hours and disappeared spontaneously over an average of 8 days (range: from about 2 hours to 10 days). Anamnesis indicated a high-fat intake during the preceding days in all cases and 7 from literature found in the Medline databases. Recurrences were noted in 10 patients. Thoracic duct imaging was performed in all cases to detect abnormalities or extrinsic compression as well as to eliminate differential diagnoses.

A painless, fluctuating, noninflammatory, and recurrent swelling of the left supraclavicular fossa in patients evoking an intermittent obstruction of the terminal portion of the thoracic duct was identified. A low-fat diet was found as safe and effective treatment.

## 1 Introduction

The thoracic duct is the largest lymph duct in the human body which collects lymph from not only the lower limbs and digestive structures but also the left side of the trunk and neck. The thoracic duct extends superiorly in the posterior mediastinum to collect thoracic lymph via posterior intercostal channels to empty into the junction of the left subclavian and jugular veins.^[1]^ There is considerable variability in the lymphatic anatomy in humans, particularly in the thoracic duct.

Even though it is rare, lymphoceles or cysts on the terminal part of the duct can develop and become permanent supraclavicular masses.^[2,3]^ The pathogenesis of these cysts includes congenital weakness in the duct wall, an acquired inflammatory process, as well as the postoperative or traumatic disruption of lymphatic drainage.^[4]^ When supraclavicular cysts are persistent, patients are most often referred to a surgeon who will discuss surgical intervention which most often results in thoracic duct ligation and includes lymphovenous anastomosis.^[5–9]^ Less frequently, paroxysmal cysts of the terminal part of the duct appear as a renitent mass above the left clavicle.^[10–12]^ Diagnosis can be difficult in these cases and physical examinations or imaging might be performed after the mass has disappeared. The aim of this study was to describe and review cases of recurrent lymphangiectasia of the left supraclavicular fossa. The epidemiological, clinical, evolutionary, and therapeutic aspects of this rare occurrence were also analyzed. Cases were collected according to 2 methods. The first was a case collection from memory from the centers of Lyon, Clermont-Ferrand, and Le-Puy-En-Velay from September 2010 to October 2020. The second was completed with a systematic review of the literature.

## 2 Methods

The Departments of Internal Medicine at Le-Puy-En-Velay, Lyon, and Clermont-Ferrand were first contacted to collect memory observational cases of recurrent thoracic duct cyst of the left supraclavicular fossa. This initial process resulted in 6 observations from September 2010 to December 2020. Information on the study and its written consent was sent to all patients involved. Institutional review board of the hospital of Le-Puy-En-Velay approved the study.

The literature review was based on a search for articles concerning intermittent terminal thoracic duct cysts that were published before December 31, 2020 and performed according to the PRISMA guidelines.^[13]^ The search parameters were limited to Medline databases which included PubMed and the National Library of Medicine in Bethesda, MD. Publication titles and abstracts were initially screened followed by a reading of the full applicable texts. Original papers published in English or French were eligible for inclusion.

The following PubMed search strategy was used: ((thoracic duct [MeSH Terms]) AND (cyst [MeSH Terms])) OR (lymphangiectasia [MeSH Terms]). All articles were selected without time restrictions. Reference lists of articles were scanned for references not initially identified in the primary search. No exclusions for study type, sample size, or setting were applied. The extracted variables consisted of the type of study, age, gender distribution, disease duration, recurrences (both in number and frequency), complications, triggering factors, treatment, and the duration of follow-up. Studies fulfilling these inclusion criteria were reviewed by all authors.

All titles that were irrelevant to this study were excluded during the first identification phase. Only articles that contained clinical data about recurrent thoracic duct cysts around the left supraclavicular fossa were retained. Abdominal or intestinal cysts, as well as permanent supraclavicular thoracic duct cysts were rejected. Studies containing only therapeutic or radiological data were not considered for the following case descriptions. Risk of biases was not evaluated because of all the studies were case reports.

The study was registered on https://www.crd.york.ac.uk/prospero/, (number CRD42021246163) and the protocol was not prepared. Authors declare no conflict of interest.

## 3 Results

Six observational cases were identified by contacting the internal medicine departments of 3 hospitals in southeastern France (Le-Puy-en-Velay, Lyon, and Clermont-Ferrand) from September 2010 to December 2020. The following are descriptions of these original series (n = 6) of observational cases 1 to 6.

### 3.1 Observational case study 1

**Figure 2:**
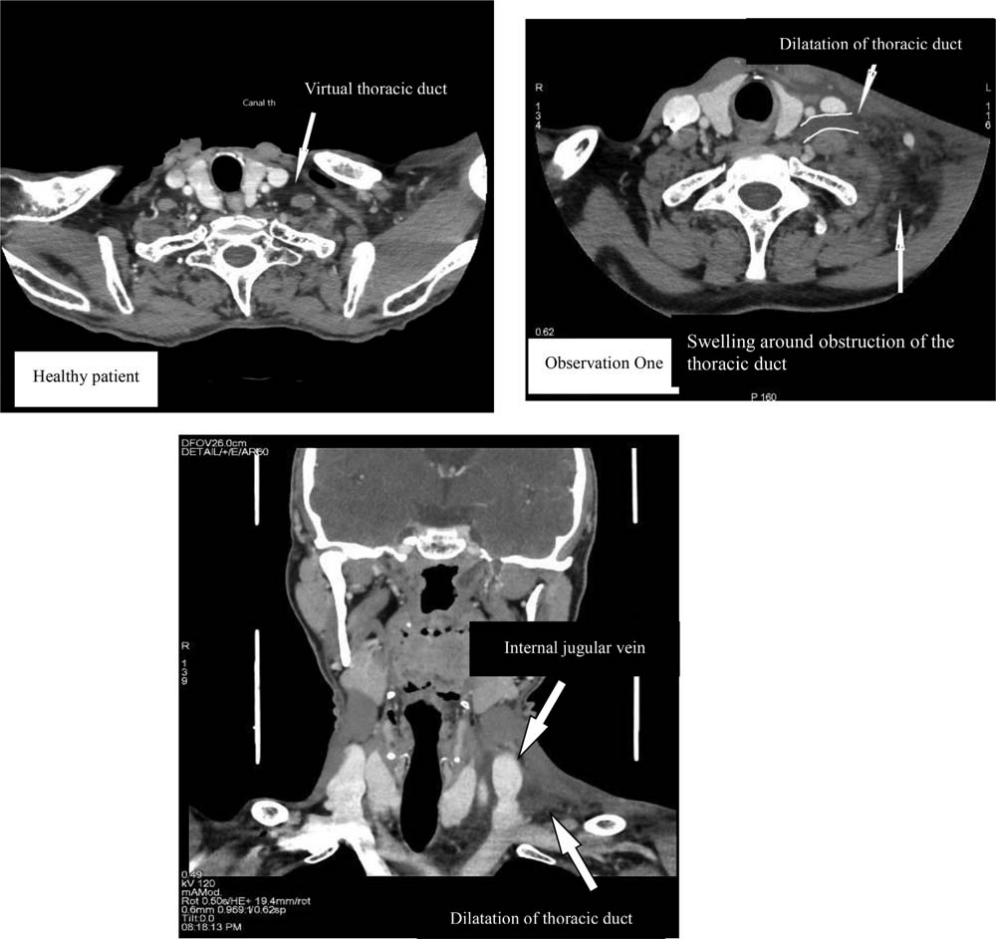
Comparison between scannographic slides of observation 1 and a healthy patient showing obstruction and dilatation of the terminal portion of the thoracic duct with swelling around the dilatation.

**Figure 3:**
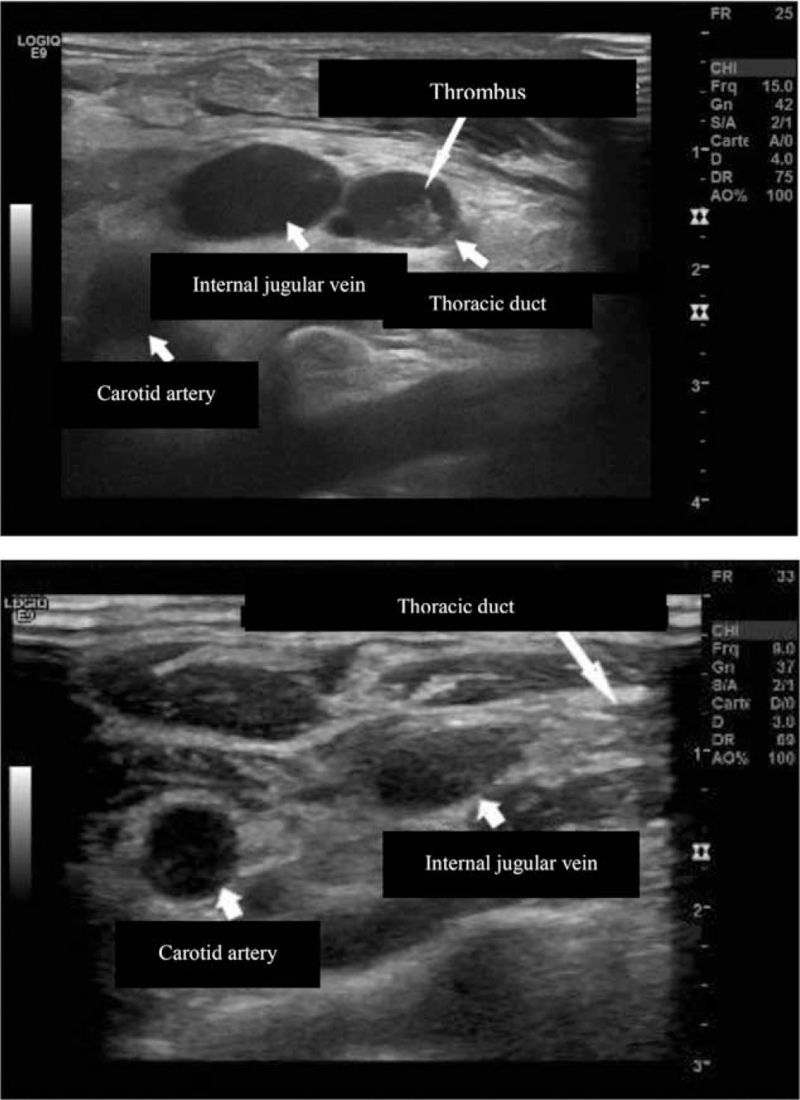
Ultrasonography sections of a patient in observation 1 (top) versus a healthy patient: dilated thoracic duct with holding an echogenic structure (thrombosis).

A 50-year-old man with a medical history of surgery for an inguinal hernia was urgently admitted for a left supraclavicular swelling which appeared when he woke up the same morning. He was not taking any medication and there was no history of occupational exposure. The mass was neither painful nor inflamed and subsequent laboratory tests were clear. A computed tomography (CT)-scan showed an infiltration of the soft tissue located on the left side of the neck and in the posterior mediastinum. The left internal jugular vein appeared tubular which suggested thoracic duct thrombosis. There was also an upstream expansion associated with a minor pleurisy on the left side (Fig. 2). A Doppler ultrasound revealed a partial occlusion of the thoracic duct due to a lipid clot (Fig. 3). The patient had consumed an entire bowl of olive oil the day before he was admitted to hospital. The outcome was spontaneously favorable after 72 hours and there was no recurrence.

### 3.2 Observational case study 2

A 74-year-old woman was admitted to the hospital because of a sudden swelling of the left supraclavicular fossa. She had a history of high blood pressure and hypothyroidism due to a partial thyroidectomy. She had taken L-thyroxin and an angiotensin-converting enzyme inhibitor. One year prior, she suffered from a similar episode which resolved itself within a few days without treatment. A high-fat meal and alcohol intake preceded both of these events. Laboratory tests were normal, however, a CT-scan showed a minor bilateral pleural effusion (predominant on the left side), an infiltration of the left side of the neck expanding to the left hollow armpit and the mediastinum, several supraclavicular adenomegalies, and an unraveled appearance of the left internal jugular vein which was deemed congruent with a possible thrombosis. An anticoagulant therapy was therefore started. Doppler ultrasound was performed 72 hours after admission and did not reveal any thrombosis and treatment was discontinued. The patient's outcome was spontaneously favorable in 24 to 48 hours and a CT-scan was subsequently performed later which came back normal.

### 3.3 Observational case study 3

A 45-year-old woman was seen in the internal medicine unit complaining of pain in the left supraclavicular fossa. She had a history of right shoulder surgery for calcifying tendinopathy following corticoid infiltration and she complained of headaches. She was using a copper intrauterine device but was not taking any medication. For a few months, she experienced night sweats without any weight loss and for 1 month, she suffered from a painful swelling of her left supraclavicular fossa as well as difficulties in swallowing. Fifteen days after the onset of these symptoms, she had a fever lasting 24 hours. Ultrasonography was performed which showed inflamed and infiltrated subcutaneous tissue on the supraclavicular fossa and on the omohyoid muscle. A CT-scan confirmed the findings and also indicated an extended inflammatory infiltrate in the retroperitoneum. Laboratory tests were normal. During examination, the patient was in good general condition; however, there was a swollen and painful mass on her left supraclavicular fossa. During an abdominal examination, there was pain in the right hypochondrium and iliac fossa. The outcome was spontaneously favorable within 48 to 72 hours. A magnetic resonance imaging (MRI) scan of the thoracic duct showed a small cyst on the cervical portion. After 3 years of follow-up, no recurrence occurred.

### 3.4 Observational case 4

**Figure 4:**
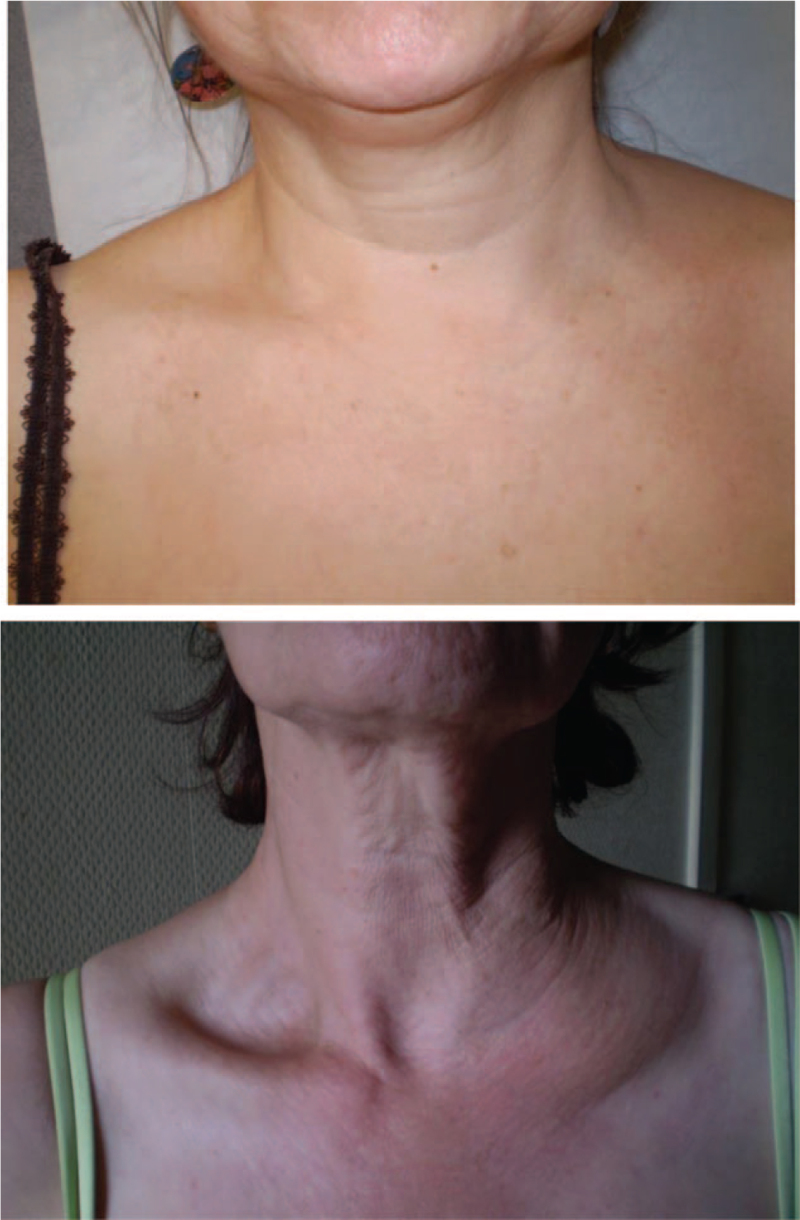
Photos of patients in observation 4 and observation 5 showing the supraclavicular mass demonstrating the obstruction of the final portion of the thoracic duct.

A 65-year-old woman was seen in the internal medicine unit for an intermittent swelling of her left supraclavicular fossa located on a preexisting area of chronic infiltrations (Fig. 4). Her medical history included hypertension, acid reflux due to a hiatal hernia, appendectomy, and spinal arthrodesis. A beta-blocker, antacid, and benzodiazepine were taken as daily medications. No triggering factor could be found for the intermittent swelling which could last from a few hours to a few days. Some of these episodes could be associated with dyspnea. Functional and ponderal C1 inhibitors were normal. There was no deficiency or C1q and monoclonal gammapathy nor antinuclear antibodies existed. A CT-scan and ultrasonography came back normal. A diagnosis of thoracic duct dysfunction was confirmed by lymphoscintigraphy which showed an impregnation delay of 2 hours and 45 minutes on the axillary lymphatic vessel (normal values are generally between 10 and 30 minutes)^[14]^ on both sides with the left side impregnation being the slowest. There was also a 14 mm adenopathy. Simple monitoring was proposed.

### 3.5 Observational case 5

A 64-year-old woman was referred for an internal medicine consultation for reoccurrence of diffuse abdominal pain associated with a left supraclavicular edema (Fig. 4). Her medical history consisted of a herniated disk, an appendectomy, and subclavicular vein thrombosis during pregnancy (at the age of 38) which was treated with 6 months of anticoagulant. She was not taking any medication. The patient indicated that a similar event had occurred 1 year before and that a Doppler ultrasound had been performed which indicated a condition consistent with a partial thrombosis of the left subclavicular vein. However, a novel imaging performed 48 hours later negated the previous diagnosis Her subsequent CT scan showed a liquid infiltration of the left supraclavicular hollow in the retroperitoneum and also in the mediastinum. Laboratory tests showed a normal functional and ponderal dosage of C1 inhibitor and serum complement level. TSH and albumin test results were normal and autoimmunity was negative. A few weeks after, a CT-scan was repeated and failed to find any abnormality. At the consultation, she once again exhibited edema in the left supraclavicular fossa and abdominal pain. These symptoms disappeared 24 hours later. Biological tests (lactate dehydrogenase [LDH], D-dimers, serum level complement, and a search for paroxysmal nocturnal hemoglobinuria) were unnoticeable. Another CT-scan was performed which revealed the same liquid infiltration found during the first episode. A significant delay of the lymphatic flow in the thoracic duct was confirmed by lymphoscintigraphy. In light of these repeated symptomatic episodes, dietary intervention was proposed with a long-chain lipid low diet, combined with medium-chain triglyceride supplementation with grape seed oil, retinyl palmitate, and tocopheryl acetate. Over the next 3 years, only 1 recurrence of swelling was reported. The patient was asymptomatic except for an inconsistent feeling of tension in her left supraclavicular fossa without any noticeable swelling being noticed during clinical examination.

### 3.6 Observational case 6

A 62-year-old woman was admitted for a left supraclavicular renitent mass that had appeared 3 weeks prior. She indicated suffering from chest and abdominal pain during the preceding 24 hours. Her medical history consisted of surgery on 2 benign thyroid nodes. Her father had had pancreatic cancer and her 2 grandmothers had died of digestive related cancers. She was not taking any medication. A CT-scan was performed showing a left supraclavicular mass associated with abdominal infiltration distributed around the colon as well as minor bilateral pleural effusions. Laboratory tests were inconclusive and there was an absence of any inflammatory syndrome. Renal and hepatic functions as well as blood counts were normal. A pleural puncture was performed and the analysis of the liquid yielded a chylous effusion. A Position Emission Tomography Scan which looked for a solid tumor or hemopathy did not show any uptake. A low-fat diet was introduced and the symptoms disappeared within 48 hours. Pulmonary radiography executed 10 days after hospital admission showed a minor right pleural effusion even though the patient was asymptomatic. Patient questioning confirmed substantial fat intake and alcohol consumption the day before the symptoms appeared. A medium chain triglyceride supplementation regime was started. No recurrence was observed 3 months after.

### 3.7 Literature search

**Figure 1:**
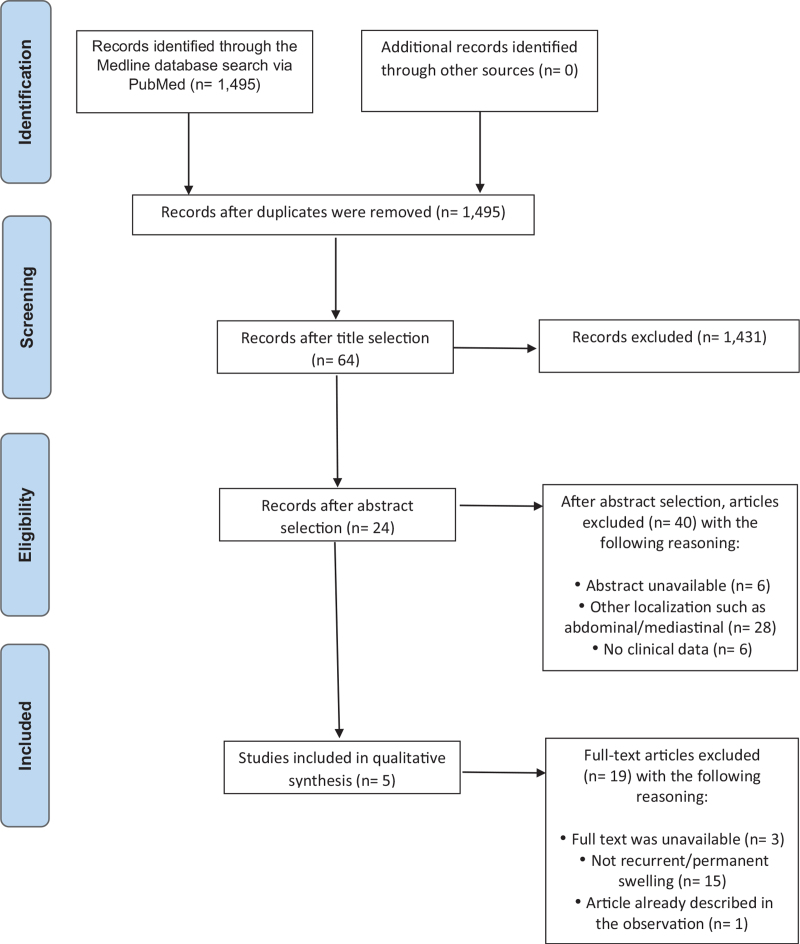
Study flow chart.

Overall, 5 original articles were included in this study. The screening process is shown in Figure 1. Our research identified 1495 articles through the Medline database search via PubMed. We checked for duplicates and 64 articles were selected according to their titles. Eligibility of articles was established after reading their abstracts. Six abstracts were unavailable; 28 articles were about abdominal or mediastinal location of duct cyst and another 6 had no clinical data. In the end, twenty-four articles were then selected with 5 qualitative studies included (3 studies had no available text, 15 articles focused on permanent and no recurrent cyst, and 1 article described a case already included in 1 of the observational case reports.

Our literature review identified 8 cases:

1. Dendorfer et al described a case of a 33-year-old woman with a recurrent thoracic duct cyst for 5 days with several occurring events within 3 years. These events were favorized by fat-rich meals and alcohol consumption. Symptoms included a non-painful mass in the left supraclavicular fossa associated with diffuse abdominal, flank tenderness, fullness, increase in waist circumference, and a weight gain of 1 to 2 kg. Diagnosis was established by echography and confirmed by a fluid punction in acute phase. Abdominal examinations eliminated ascites, pleural effusion, and lymphadenopathy.^[12]^
2. Veziant et al reported a 49-year-old woman with an acute mass in the neck with respiratory discomfort and lateral chest pain. Diagnosis of the duct cyst in the left supraclavicular fossa was associated with chylothorax and chyloperitoneum and was demonstrated by fluid punction with symptoms lasting for 10 days. The patient followed a low-fat diet however recurrence appeared 10 years later and led to the patient to have surgery.^[9]^
3. Preyer et al identified 4 premenopausal women (aged 22–48 years) with recurrent duct cysts of the left supraclavicular fossa. The first case had recurrent swelling for 3 months and the diagnostic was established by echography, MRI and surgery (lymphatic fluid in the cyst). The second case patient had a recurrent mass in the left supraclavicular fossa for 7 months corresponding to a duct cyst after being detected with an echography, RMI, and surgery. The third case had swelling in the left supraclavicular fossa for 10 years. Diagnosis of the duct cyst was made by echography, tomodensitometry, and surgery. The last case consulted for a mass in the left supraclavicular fossa for several weeks. Echography, tomodensitometry, and surgery made the diagnostic of these recurrent thoracic duct cysts. All 4 patients did not have a significant medical history and took estrogen-containing medication at the onset of neck mass enlargement. Surgery was performed because the cyst was cosmetically disturbing (mass were noninflammatory). Recurrence after surgery only appeared in the fourth case.^[16]^
4. Zatterstrom et al reported the follow-up of a 55-year-old man who was consulted 25 years ago for a fluctuant supraclavicular mass that appeared a few months before. The diagnosis was established by tomodensitometry, fluid puncture, and pedal lymphography. No treatment was made. Nine years after, there was a progression of the cyst, followed by the cyst diminishing spontaneously 13 years later and disappearing 25 years after. Retrospectively, it was reported that a high fat diet triggered the swelling.^[15]^
5. Van Den Bussche et al described the case of a patient with swelling on the left side of the neck spontaneously appear (without discomfort or pain, compressible and nonpulsatile). This patient was an 83-year-old woman. Echography, tomodensitometry, and puncture confirmed duct cyst. Moreover, the cyst was punctured and was kept on a medium chain triglyceride diet. No recurrence appeared at the follow-up (1 month later).^[4]^

The data from 8 external cases along with cases from our institutions are shown in Table 1. One patient's case study (observational case study 5) has been published elsewhere.^[11]^ The authors of the 4 articles were mostly surgeons^[4,9,15,16]^ and the remaining article was written by a radiologist.^[12]^

**Table 1:** Overview of our cases and studies assessing patients with transient obstruction of the thoracic duct terminal portion.

Studies	Sex	Age	Pathology duration	Episode duration	Recurrences	Complications	Trigger	Treatment	Follow up
Dendorfer et al^[12]^	F	33	3 yr	5 d	Several	No	Fat/Alcohol	DI	No follow up
Veziant et al^[9]^	F	49	10 yr	10 d	2 over a 10-yr period	Chylothorax and Chyloperitonéum	Fat/Alcohol	DI then 10 yr after LVA	No reccurrences in 3 yr after LVA
Preyer et al^[16]^	F	36	2–3 mo	NK	Several	No	Clomiphène	Surgery	No
Preyer et al^[16]^	F	37	7 mo	NK	Several	No	COC	Surgery	1 recurrence on the right side
Preyer et al^[16]^	F	48	10 yr	NK	Several	No	Estradiol valerate	Surgery	No
Preyer et al^[16]^	F	22	Few weeks	10 d	2	No	COC	Surgery	No
Zatterstrom et al^[15]^	M	55	24 yr	Few years	0	No	High fat diet	DI	10 yr
Van Den Bussche et al^[4]^	F	83	1 mo	1 wk	1	No	Hypertriglyceridemia	Puncture and DI	No
Observational study case 1	M	50	1 mo	3 d	1	No	Fat	DI	No
Observational study case 2	F	74	1 yr	1–2 d	2	No	Fat/Alcohol	DI	No
Observational study case 3	F	45	1 mo	1 mo	1	Pain	Fat/Alcohol	DI	No reccurrences in 3 yr
Observational study case 4	F	65	7 yr	Few hours to few days	Several	No	NI	DI	No
Observational study case 5	F	64	10 yr	1 d	4	Pain	Pregnancy, then NI	DI + LIPROCIL	1 reccurence in 2 yr
Observational study case 6	F	62	3 wk	3 wk	2	Chylothorax + Chyloperitoneum	Fat/alcohol	DI + Nutricia	No recurrence in 2 wk

COC = combined oral contraceptive, DI = dietary intervention, LVA = lymphovenous anastomosis, NI = not identified, NK = not known.

Studied patients were predominately female (12 out of 14 patients, sex ratio: 6) with only 1 case from the literature search described as a male and 1 man was included in our case reports. The mean age of patients included in this study was 51 years old (median, 49; range, 22–83 years) and there was no difference between our data and the literature found on Medline. All patients presented a painless mass of the left supraclavicular fossa. The mass was always noninflammatory, renitent, transient, nonpulsatile, and compressible. Palpation of the mass was painless and compressible. Clinical examinations between relapses were strictly normal.

Two cases were associated with ascites and pleuritis.^[9]^ The mean duration of the episodes was 8.4 days and ranged from a few hours to 1 month with a median of 9 days (Q25%: 2.5 days; Q75%: 10 days). The evolution duration of the mass could only be included in 9 cases because Preyer et al did not specify this data, and Zatterström described a fluctuating mass (male patient) that had existed for 25 years and had grown constantly until a change in diet made it disappear.^[15,16]^

### 3.8 Triggering factors

A triggering factor was identified in 12 of the 14 cases. Preyer et al assumed the intermittent obstruction of the terminal portion of the thoracic duct was due to an estrogen pill, but the article did not specify data related to the patient's diet prior to the appearance of the swelling.^[16]^ Two other cases showed a high-fat diet was consumed a few days before the episodes. Zatterström et al described the disappearance of the supraclavicular mass after the patient initiated a change in diet.^[15]^ In the cases handled by the authors of this study, a high-fat diet has been clearly identified in 4 cases and was strongly suspected in the 2 other cases since dietary intervention made it possible to resolve the symptoms.

### 3.9 Imaging and complementary tests

All patients underwent an ultrasound. Tomodensitometry was used in 13 cases, with an MRI in 3, and positron emission tomography scan was done for 1 case. Lymphoscintigraphy was performed in 4 cases and showed an increased delay of impregnation on the axillary lymphatic vessel. During the acute phase, ultrasound imaging showed infiltrated subcutaneous tissue with a hypoechoic mass with liquid in the left supraclavicular fossa in some cases.^[9,12]^ Tomodensitometry showed an inconsistent cystic mass in the left supraclavicular region combined with an infiltration of the soft tissue in the left side of the neck.^[9,16]^ Tomodensitometry could also have shown pleural effusion or ascites.^[9]^ All subsequent examinations were normal following the acute phase. Laboratory tests were deemed normal and a lack of any inflammatory syndrome was evident.

In 3 cases found in the literature search,^[4,9,12]^ a puncture of the mass was performed but none were done in any of the cases in our study. In 4 cases, no puncturing occurred because ablation was directly performed. In the observational study case of the supraclavicular mass, which was associated with pleuritis, Veziant et al completed pleural puncturing because it was more easily carried out than a puncture procedure. When punctures of mass or pleura were accomplished, a milky fluid that contained numerous lymphocytes (98%–100% ^[4,12]^), low albumin level (2.3–3.6 mg/dL ^[4,12]^), high triglycerides levels (1579–2950 mg/dL ^[9,12]^), and no abnormal cells^[4,9,12]^ was found in all subjects. The biochemical analyses of this liquid were in favor of chyle.

### 3.10 Follow-up

The duration of patient follow-up beginning with the detection of the renitent mass to diagnosis varied greatly with a mean duration of 4.7 years (range, 3 weeks up to 25 years). Follow-up after therapeutic intervention depended on the proposed and short follow-ups were justified because 4 patients had undergone surgery quickly after diagnosis.^[16]^ Van den Bussche et al described a case without reoccurrence and the follow-up lasted only 1 month.^[4]^

### 3.11 Treatment

In 4 cases surgery was performed quickly after diagnosis.^[16]^ Eight cases received a low-fat diet alone including 2 cases of literature,^[12,15]^ In 1 case a no-fat diet was proposed first and surgery was done after the first recurrence 10 years later.^[9]^ In 1 case, patient had a low-fat diet associated with a mass puncture without recurrence but the follow-up was short (1 month).^[4]^ In our subjects, dietary intervention was the only treatment and all the patients were advised on dietary rules with 2 benefiting from a strict diet of light-chain triglyceride removal. No complication was described in the case of low-fat diet. Preyer et al reported a surgical complication related to a chylous fistula.

## 4 Discussion

This study comprised the largest set of patients with recurrent lymphangiectasia of the left supraclavicular fossa. This disorder mostly affects middle-aged women and appears as a left supraclavicular mass which is neither inflammatory nor renitent. It is transient and regresses in an average of 8 days. A recent high-fat diet is a major triggering factor. In the acute phase, lymphoscintigraphy remains the benchmark because it can reveal thoracic duct obstruction; however, this procedure is increasingly being replaced by noninvasive tests. Imaging should be repeated after the acute episode to eliminate differential diagnoses. A low-fat diet is frequently proposed and it is most often effective in preventing recurrences.

Overall, despite some differences regarding clinical and imaging features, the stereotypical clinical history in all cases suggests the same disease. No large-scale studies describing clinical, imaging, prognosis, or treatment parameters were found in this study. Moreover, this disease has probably been underestimated and underdiagnosed since only 6 observed cases at 3 internal medicine departments were found over a 10-year period in the medical facilities. It should, however, remain a diagnosis of exclusion and it may be therefore crucial to exclude differential diagnoses such as a tumor-like metastatic lesions, a parathyroid or thymic cyst,^[6–8,17–19]^ sequelae of a prior injury (whether surgical or other including birthing, whiplash and stretching),^[8,15,18]^ lymphangioma^[6,17,19]^ or hematoma.^[11]^ In these cases, a clinical examination and medical imaging during the acute phase should guide a clinician. The most plausible explanations for this disorder are a weakness of the thoracic duct wall.^[2,4,16]^ This weakness can be congenital, related to an inflammatory process, or stem from an anatomical conflict at the angle between the left intern jugular vein and the left subclavicular vein.

In most of our patients, triggering factors were a high-fat diet and combined oral contraceptive use.^[1,16,17,20]^ It is important to note that the cases involved combined oral contraceptive use dated from 1995 which was at a time when the average dosage of estrogen was high. A high-fat diet can be a triggering factor because it increases pressure in the thoracic duct canal^[1]^ with the resulting production of chyle that is composed mainly of lymphocytes and lipid-rich fats.^[1]^ The designation “Nutella Syndrome” that was found in 1 publication has sometimes been used because of the link between recurrences after consumption of a high-fat meal^[20]^ and we propose to name it.

Thoracic duct imaging is decisive because it reveals secondary causes of dilatation and/or malformation such as lymphangioma, stenosis of the terminal valve of the thoracic duct, extrinsic compression, and venous thrombosis, all of which require specific treatment plans.^[9,10]^ Doppler ultrasonography should be carried out first in the case of swelling of the supraclavicular fossa since it is noninvasive and can be performed quickly.^[7,20]^ It may show a cystic lesion, hypoechogenic to echofree mass, with no connections to vessels, sono compressible, sharply outlined, with no internal perfusion.^[2,4,9,10,12,18]^ In the acute phase, tomodensitometry can show a dilation of the thoracic duct or cysts located adjacent to the left internal jugular vein.^[15,18]^ Cervical soft tissue surrounding the cyst can appear infiltrated with edematous fluid.^[12]^ The mass is hypoechoic, homogenous, thin-walled, and emit water attenuation signal inside.^[5–7]^

An MRI can highlight bright lesions on the T1 and T2-weighted imaging results arising from the thoracic duct close to the left subclavian vessels,^[6,18]^ sometimes with dilated a thoracic duct and apparent communication of the cyst with the thoracic duct.^[3,7]^ Repeating radiological examinations after the acute phase is important because an edema present in the initial phase may mask abnormalities of the terminal portion of the thoracic canal.^[7,9]^ In the case of the “Nutella Syndrome,” imaging taken after the acute crisis must be normal. Lymphoscintigraphy is the benchmark because it provides a proper analysis of the shape of the thoracic duct and can measure its drainage rate.^[9,14,19,21]^ Today, CT-scans and MRIs are more commonly used.

Lymphoscintigraphy is time-consuming, invasive, and can be difficult to interpret in the absence of standardized procedures^[2,21]^ since normal diffusion time values are from 10 to 30 minutes and considered delayed after 120 minutes.^[14]^ In case of dilation of the thoracic duct, it increased delay of diffusion up to the thoracic duct, without extravasation.^[3,15,18]^

In doubt of diagnosis, a puncture to the cyst can be performed to indicate a chyle-like liquid composed of 90% to 100% lymphocytes and rich in triglycerides.^[3,17]^ When the cyst is removed, immunohistochemical analysis can show CD31 CD34 factor VIII and keratin positivity which are biomarkers of the epithelial and endothelial nature of a wall vessel, thus removing any doubt about cancer or a lymphocele.^[4,7,12,15,22]^ Complications can occur when cysts become permanent and cause compression of the surrounding anatomical structures.^[7,9,10]^

Therapeutic treatment management is not well defined and should be guided by the patient's symptoms. According to the symptomatology, a restricted diet should be discussed first. The diet should exclude food rich in fat. If long-chain triglycerides are completely excluded from the diet, medium-chain triglyceride supplementation should be introduced^[4,17,23]^ because medium-chain triglycerides are directly absorbed into the portal system.^[1,23]^ In our study, a dietary regime with or without total exclusion of long-chain triglyceride was offered systematically as a first step because it is safe and usually enough to control the disease. Surgical intervention is rarely considered and should be reserved for highly symptomatic and serious cases.

Our study had several strengths. To our knowledge, it is the first literature review conducted on this disease. Of the 14 described cases, we reported 6 which had follow-ups on clinical outcomes allowing a better description of the pathology. Limitations were related to the retrospective nature of the study and lack of subsequent data. A potential recall bias may have occurred because some observations were solely based on 1 doctor's remembrance. As previously discussed, this disease is rare and potentially underdiagnosed. The prevalence of this disease is still unknown and other studies are required to determine its overall prevalence, clinical presentation, evolution, and pathophysiology with more accuracy.

## 5 Conclusion

An intermittent swelling of the left supraclavicular fossa should raise suspicion that an intermittent cyst may be in the terminal portion of the thoracic duct. It is important to look for any unusual intake of high-fat foods preceding symptoms. Thoracic duct imaging is crucial to eliminate differential diagnoses. Lymphography remains the benchmark for diagnosis, however, noninvasive imaging technics (MRI or CT-scan) are currently preferred. In most cases, therapeutic management is a conservative way to manage this disease with dietary intervention consisting of a low-fat diet.

## Author contributions

**Conceptualization:** Julie Planchette, Benjamin Castagne, Anne-Sophie Resseguier, Pascal Seve.

**Investigation:** Julie Planchette, Clara Jaccard, Audrey Nigron, Jean-Baptiste Chadeyras, Guillaume Le Guenno, Benjamin Castagne, Anne-Sophie Resseguier, Pascal Seve.

**Methodology:** Julie Planchette.

**Supervision:** Yvan Jamilloux, Pascal Seve.

**Writing – review & editing:** Yvan Jamilloux, Pascal Seve.
